# Supplementary material for: Minimization of the Bacillus subtilis divisome suggests FtsZ and SepF can form an active Z-ring, and reveals the amino acid transporter BraB as a new cell division influencing factor
Source: PLoS Genet. 2025 Jan 27;21(1):e1011567. doi: 10.1371/journal.pgen.1011567 (PMC11790237; doi:10.1371/journal.pgen.1011567)
Supplement: S1 Appendix — This file describes the construction of BMD and other mutants listed in Table A (B. subtilis strains used in this study), and the primers used for the construction, listed in Table B (Primers used in this study). It includes the following supplementary figures: Fig A: Gene deletion pathways, Fig B: Growth of Bacillus minimal divisome strains, Fig C: FtsZ and nucleoid localization in BMD27, Fig D: FtsZ and nucleoid localization in ftsA::erm, Fig E: BMD27 in stationary phase, Fig F: Phenotype of the braB deletion mutant, Fig G: Sensitivity of ΔbrnQ and ΔbcaP mutants for 3-MBA, Fig H: Effect of membrane fluidity on sensitivity for FtsZ perturbations, and a list of References. (PDF) [file pgen.1011567.s001.pdf]

S1 Appendix

Minimization of the *Bacillus subtilis* divisome suggests FtsZ and SepF can form an active Z-ring, and reveals the amino acid transporter BraB as a new cell division influencing factor

Ilkay Celik Gulsoy, Terrens N. V. Saaki, Michaela Wenzel, Simon Syvertsson, Taku Morimoto, Tjalling K. Siersma, Leendert W. Hamoen

- Construction of BMD mutants
- Construction of other mutants
- Table A *B. subtilis* strains used in this study
- Table B Primers used in this study
- Fig A Gene deletion pathways
- Fig B Growth of Bacillus minimal divisome strains
- Fig C FtsZ and nucleoid localization in BMD27
- Fig D FtsZ and nucleoid localization in *ftsA::erm*
- Fig E BMD27 in stationary phase
- Fig F Phenotype of the *braB* deletion mutant
- Fig G Sensitivity of Δ*brnQ* and Δ*bcaP* mutants for 3-MBA
- Fig H Effect of membrane fluidity on sensitivity for FtsZ perturbations
- References

S1 Appendix

Construction of BMD mutants

The minimal divisome mutants were constructed using a marker-free deletion method described by Morimoto et al. [1]. A deletion cassette was constructed comprising the upstream and downstream region of the target genes, followed by the IPTG-inducible MazF toxin, a spectinomycin marker for selection and the target gene. This cassette was transformed into the natural competent (transformable) wild type *B. subtilis* 168 cells and selection on spectinomycin containing LB agar plates. After transformation of the donor DNA, the toxin was induced by IPTG, forcing the excision of the deletion cassette by intra-molecular homologous recombination, resulting in deletion of the target gene. After transformation the presence of deletions were checked with PCR. The resulting deletion strain was then used as recipient strain to introduce the next gene deletion.

To create a marker-free *zapA* deletion mutant, the upstream region of *zapA* was amplified with primer pair yshA-DF1/yshA-DR1.2, the downstream region with yshA-DF2.2/yshA-DR2, the MazF-cassette was amplified with yshA-MF/yshA-DR2a from genomic DNA of strain TMO310 [1], and *zapA* was amplified with primer pair yshA-MFa/yshA-MR. The purified PCR products were fused by overlap extension PCR using the flanking primers yshA-DF1 and yshA-MR. The purified final product was transform to competent wild type *B. subtilis* cells resulting in strain *zapA-spec*. MazF toxin induction with IPTG stimulated recombination and removal of the toxin cassette and *zapA*, resulting in strain BMD1.

To create a marker-free *minC* deletion, the upstream region of *minC* was amplified with primer pair minC-DF1/minC-DR1.2, the downstream region with minC-DF2.2/minC-DR2, the MazF-cassette with minC-MF/minC-DR2a from genomic DNA of strain TMO310 [1], and *minC* was amplified with minC-MFa/minC-MR. The purified PCR products were fused by overlap extension PCR using the flanking primers minC-DF1 and minC-MR. The purified final

product was transform to competent wild type *B. subtilis* cells resulting in strain *minC-spec*. Chromosomal DNA from this strain was transformed to BMD1, and subsequent MazF toxin induction with IPTG removed the toxin cassette and *minC*, resulting in strain BMD2.

To create a marker-free *ugtP* deletion, the upstream region of *ugtP* was amplified with primer pair ugtP-DF1/ ugtP-DR1.2, the downstream region with ugtP-DF2.2/ugtP-DR2, the MazF-cassette was amplified with ugtP-MF/ugtP-DR2a from genomic DNA of strain TMO310 [1], and *ugtP* was amplified with the primer pair ugtP-MFa/ugtP-MR. The purified PCR products were fused by overlap extension PCR using the flanking primers ugtP-DF1 and ugtP-MR. The purified final product was transform to competent wild type *B. subtilis* cells resulting in strain *ugtP-spec*. Chromosomal DNA from this strain was transformed to BMD2, and subsequent MazF toxin induction with IPTG removed the toxin cassette and *ugtP*, resulting in strain BMD3.

To create a marker-free *minJ* deletion, the upstream region of *minJ* was amplified with primer pair minJ-DF1/minJ-DR1.2, the downstream region with minJ-DF2.2/minJ-DR2, the MazF-cassette was amplified with minJ-MF/minJ-DR2a from genomic DNA of strain TMO310 [1], and *minJ* was amplified with the primer pair minJ-MFa/minJ-MR. The purified PCR products were fused by overlap extension PCR using the flanking primers minJ-DF1 and minJ-MR. The purified final product was transform to competent wild type *B. subtilis* cells resulting in strain *minJ-spec*. Chromosomal DNA from this strain was transformed to BMD3, and subsequent MazF toxin induction with IPTG removed the toxin cassette and *ugtP*, resulting in strain BMD5.

To create a marker-free *ezrA* deletion, the upstream region of *ezrA* was amplified with primer pair ezrA-DF1/ezrA-DR1.2, the downstream pair with ezrA-DF2.2/ezrA-DR2, the MazF-cassette was amplified with ezrA-MF/ezrA-DR2a from genomic DNA of strain TMO310 [1],

and *ezrA* was amplified with the primer pair ezrA-MFa/ezrA-MR. The purified PCR products were fused by overlap extension PCR using the flanking primers ezrA-DF1 and ezrA-MR. The purified final product was transform to competent wild type *B. subtilis* cells resulting in strain *ezrA-spec*. Chromosomal DNA from this strain was transformed to BMD5, and subsequent MazF toxin induction with IPTG removed the toxin cassette and *ugtP*, resulting in strain BMD6.

To create a marker-free *spx* deletion, the upstream region of *spx* was amplified with primer pair spx-DF1/spx-DR1.2, the downstream region with spx-DF2.2/spx-DR2, the MazF-cassette was amplified with primerset spx-DR2a/spx-MF from genomic DNA of strain TMO310 [1], and *spx* was amplified with the primers spx-MFa and spx-MR. The purified PCR amplicons were fused by overlap extension PCR with the flanking primers spx-DF1 and spx-MR. The purified final product was transform to competent wild type *B. subtilis* cells resulting in strain *spx-spec*. Chromosomal DNA from this strain was transformed to BMD6, and subsequent MazF toxin induction with IPTG removed the toxin cassette and *ugtP*, resulting in strain BMD7.

To create a marker-free *clpX* deletion, the upstream region of *clpX* was amplified with primer pair clpX-DF1/clpX-DR1.2, the downstream region with clpX-DF2.2/clpX-DR2, the MazF-cassette was amplified with clpX-MF/clpX-DR2a from genomic DNA of strain TMO310 [1], and *ezrA* was amplified with the primer pair clpX-MFa/clpX-MR. The purified PCR products were fused by overlap extension PCR using the flanking primers clpX-DF1 and clpX-MR. The purified final product was transform to competent wild type *B. subtilis* cells resulting in strain *clpX-spec*. Chromosomal DNA from this strain was transformed to BMD7, and subsequent MazF toxin induction with IPTG removed the toxin cassette and *clpX*, resulting in strain BMD9.

To create a marker-free *noc* deletion, the upstream region of *noc* was amplified with primer pair noc-DF1/noc-DR1.2, downstream with noc-DF2.2/noc-DR2, the MazF-cassette was amplified with noc-MF/noc-DR2a from genomic DNA of strain TMO310 [1], and *noc* was amplified with the primer pair noc-MFa/noc-MR. The purified PCR products were fused by overlap extension PCR using the flanking primers noc-DF1 and noc-MR. The purified final product was transform to competent wild type *B. subtilis* cells resulting in strain *noc-spec*. Chromosomal DNA from this strain was transformed to BMD9, and subsequent MazF toxin induction with IPTG removed the toxin cassette and *clpX*, resulting in strain BMD12.

Subsequent removal of *ezrA* from BMD12 was achieved by transforming this strain with chromosomal DNA from strains LH69, which contains the *ezrA::tet* deletion cassette, resulting in BMD14.

The final removal of *ftsA* from BMD14 was achieved by transforming this strain with chromosomal DNA from strains LH75, which contains the *ftsA::erm* deletion cassette, resulting in BMD27. The *ftsA::erm* deletion came from strain YK206 [2], and contains a Pspac promoter that drives expression of *ftsZ*. The promoter does not require IPTG because the *lacI* repressor is not present.

Construction of other mutants

To visualize FtsZ using GFP in BMD27, a Pxyl-*gfp-ftsZ* reporter fusion located at the ectopic *amyE* locus was PCR amplified using primer pair TerS350/351 from strain 2020, and DpnI treated to destroy the template DNA. To reduce the chance of deletion mutant reversion and the fact that BMD27 is poorly transformable, the PCR product was first transformed into BMD14, yielding strain TNVS377. Next, genomic DNA of BMD27 was transformed into TNVS377 to delete *ftsA* resulting in strain TNVS385. The PCR product was also transformed to

wild type *B. subtilis*, resulting in strain TNVS391. To visualize FtsZ in a *ftsA* deletion strain, TNVS391 was transformed with chromosomal DNA from YK206 [2], resulting in strain TNVS553.

To investigate the localization of BraB, a C-terminal fusion with monomeric super folder GFP was constructed expressed from the ectopic *amyE* locus. To this end *braB* was amplified from genomic DNA using primer pair TerS391/TerS392 and Gibson assembled into pTNV64, which was linearized with primer pair TerS274/TerS368, resulting in plasmid pTNV111. This plasmid was transformed into *B. subtilis* 168 to give strain TNVS298. To remove the native copy of *braB* this strain was transformed with genomic DNA from TNVS34, resulting in strain TNVS308.

To assess the effect of the different spontaneous mutations in the BMD strains, the related genes were deleted. To this end, single deletion strains were obtained from the Bacillus Genomic Stock Centre (BGSC), and their chromosomal DNA transformed to *B. subtilis* 168. The resulting strains are listed in Table S1.

Table A: *B. subtilis* strains used in this study.

strain	genotype	reference
168	*trpC2*	[3]
TM0310	*aprE::spec, lacI, Pspac-mazF*	[1]
2020	*amyE::(Pxyl-gfpmut1-ftsZ spc)*	[4]
1356	*zapA-yshB::tet*	[5]
LH69	*ΔezrA::tet, amyE::Pxyl-gfp-ftsZ, spec*	Hamoen, unpublished
LH75	*lacA::tet ΔftsA::erm*	Hamoen, unpublished
YK206	*ftsA::erm* P_{spac}*-ftsZ*	[2]
BKE02340	*gltP::erm*	[6]
BKE00490	*spoVG::erm*	[6]
BKE04730	*sigB::erm*	[6]
BKE06970	*yesO::erm*	[6]
BKE12340	*uxuA::erm*	[6]
BKE13460	*rsgI::erm*	[6]
BKE13470	*sspD::erm*	[6]
BKE13490	*htpX::erm*	[6]
BKE13500	*ktrD::erm*	[6]
BKE13509	*ykzP::erm*	[6]
BKE13510	*ykzE::erm*	[6]
BKE13520	*ykrP::erm*	[6]
BKE13530	*kinE::erm*	[6]
BKE13540	*ogt::erm*	[6]
BKE15050	*ylbL(ddcP)::erm*	[6]
BKE22320	*ponA::erm*	[6]
BKE24770	*mgsR::erm*	[6]
BKE24780	*yqgY::erm*	[6]
BKE24790	*yqgX::erm*	[6]
BKE03220	*ycgO::erm*	[6]
BKE29600	*braB::erm*	[6]
BKE32040	*yuiF::erm*	[6]
BKE36460	*ywoF::erm*	[6]
BKE29530	*sppA::erm*	[6]
BKE21720	*ypmT::erm*	[6]
BKE18670	*yoaN::erm*	[6]
BKE39820	*htpG::erm*	[6]
minC-spec	*ΔminC::spec*, P_{spac}-*mazF*	this work
zapA-spec	*ΔzapA::spec*, P_{spac}-*mazF*	this work
noc-spec	*Δnoc::spec*, P_{spac}-*mazF*	this work
ugtP-spec	*ΔugtP::spec*, P_{spac}-*mazF*	this work
ezrA-spec	*ΔezrA::spec*, P_{spac}-*mazF*	this work
minJ-spec	*ΔminJ::spec*, P_{spac}-*mazF*	this work
clpX-spec	*ΔclpX::spec*, P_{spac}-*mazF*	this work
spxA-spec	*ΔspxA::spec*, P_{spac}-*mazF*	this work
BMD1	*ΔzapA*	this work
BMD2	*ΔzapA ΔminC*	this work
BMD3	*ΔzapA ΔminC ΔugtP*	this work
BMD5	*ΔzapA ΔminC ΔugtP ΔminJ*	this work
BMD6	*ΔzapA ΔminC ΔugtP ΔminJ ΔezrA*	this work
BMD7	*ΔzapA ΔminC ΔugtP ΔminJ ΔezrA ΔspxA*	this work
BMD9	*ΔzapA ΔminC ΔugtP ΔminJ ΔezrA ΔspxA ΔclpX*	this work
BMD12	*ΔzapA ΔminC ΔugtP ΔminJ ΔspxA ΔclpX Δnoc*	this work
BMD14	*ΔzapA ΔminC ΔugtP ΔminJ ezrA::tet ΔspxA ΔclpX Δnoc*	this work
BMD27	*ΔzapA ΔminC ΔugtP ΔminJ ezrA::tet ΔspxA ΔclpX Δnoc ftsA::erm*	this work

TNVS034	*braB::erm* (BKE29600 transformed to 168)	this work
TNVS083	*sftA::erm* (BKE29805 transformed to 168)	this work
TNVS112	*ponA::erm* (BKE22320 transformed to 168)	this work
TNVS114	*ycgO::erm* (BKE03220 transformed to 168)	this work
TNVS131	*ylbL(ddcP)::erm* (BKE15050 transformed to 168)	this work
TNVS132	*spoVG::erm* (BKE00490 transformed to 168)	this work
TNVS133	*ypmT::erm* (BKE21720 transformed to 168)	this work
TNVS134	*sppA::erm* (BKE29530 transformed to 168)	this work
TNVS135	*yoaN::erm* (BKE18670 transformed to 168)	this work
TNVS193	*zapA::tet* (1356 transformed to 168)	this work
TNVS275	*uxuA::erm* (BKE12340 transformed to 168)	this work
TNVS277	*brnQ::erm* (BKE26690 transformed to 168)	this work
TNVS279	*bcaP::erm* (BKE09460 transformed to 168)	this work
TNVS280	*glpT::erm* (BKE02340 transformed to 168)	this work
TNVS281	*ftsA::erm* (YK206 transformed to 168)	this work
TNVS292	*braB::erm* (BKE29600 transformed to 168)	this work
TNVS298	*Pxyl-braB-msfGFP*	this work
TNVS308	*braB::erm Pxyl-braB-msfGFP*	this work
TNVS377	*ΔzapA ΔminC ΔugtP ΔminJ ezrA::tet ΔspxA ΔclpX Δnoc* P_{xyl}*-gfp-ftsZ*	this work
TNVS385	*ΔzapA ΔminC ΔugtP ΔminJ ezrA::tet ΔspxA ΔclpX Δnoc* P_{xyl}*-gfp-ftsZ ftsA::erm*	this work
TNVS391	P_{xyl}*-gfp-ftsZ*	this work
TNVS401	*sigB::erm* (BKE04730 transformed to 168)	this work
TNVS474	*yesO::erm* (BKE06970 transformed to 168)	this work
TNVS475	*htpG::erm* (BKE39820 transformed to 168)	this work
TNVS476	*mgsR::erm* (BKE24770 transformed to 168)	this work
TNVS477	*yqgY::erm* (BKE24780 transformed to 168)	this work
TNVS478	*yqgX::erm* (BKE24790 transformed to 168)	this work
TNVS479	*yuiF::erm* (BKE32040 transformed to 168)	this work
TNVS515	*ywoF::erm* (BKE36460 transformed to 168)	this work
TNVS516	*ogt::ermI* (BKE13540 transformed to 168)	this work
TNVS517	*kinE::erm* (BKE13530 transformed to 168)	this work
TNVS518	*ykrP::erm* (BKE13520 transformed to 168)	this work
TNVS519	*ykzE::erm* (BKE13510 transformed to 168)	this work
TNVS520	*ykzP::erm* (BKE13509 transformed to 168)	this work
TNVS521	*ktrD::erm* (BKE13500 transformed to 168)	this work
TNVS522	*htpX::erm* (BKE13490 transformed to 168)	this work
TNVS523	*sspD::erm* (BKE13470 transformed to 168)	this work
TNVS524	*rsgI::erm* (BKE13460 transformed to 168)	this work
TNVS547	*Δbkd(lpdV-bkdAA-bkdAB-bkdB)::kan des::spec*	this work
TNVS553	*ftsA::erm amyE::(*P_{xyl}*-gfpmut1-ftsZ spc)*	this work
TNVS761	*Pspac-ftsZ:bleo(single crossover) ΔlacA::lacI(tet)*	this work
TNVS795	*Pspac-ftsZ:bleo(single crossover) ΔlacA::lacI(tet) des::ery*	this work
TNVS801	*Pspac-ftsZ:bleo(single crossover) ΔlacA::lacI(tet) Δbkd(lpdV-bkdAA-bkdAB-bkdB)::kan*	this work
TNVS830	*Pspac-ftsZ:bleo(single crossover) ΔlacA::lacI(tet) Δbkd(lpdV-bkdAA-bkdAB-bkdB)::kan des::ery*	this work

Table B: primers used in this study.

Primer	Sequence 5'-3' *
TerS274	CATCCTAGGAATCTCCTTTCTAGA
TerS350	CACCGCCGACATTCGCGTGGCTCCA
TerS391	AGAAAGGAGATTCCTAGGatgAAACACTCACTGCCTGTCA
TerS392	CCTGAGCCGCTTCCTGAGCCACTTATTTCATTAAGCTGTTTGGA
TerS351	GCATCAGGGCTGCGGCATCCGGA
TerS368	GGCTCAGGAAGCGGCTCAGGATCCAAAGGAGAAGAACTTTTCACTGGAGT
yshA-DF1	AAGCCCTGACAAGTACGGTG
yshA-DF2.2	gacgctccagGCCGTCAGACAACGTTTCTC
yshA-DR1.2	gtctgacggcCTGGAGCGTCAGCTTAAAGA
yshA-DR2	CTGATTGGGTAggatccgcgCCTTGAAACAAGACCTTACC
yshA-DR2a	GGTAAGGTCTTGTTTCAAGGcgcggatccTACCCAATCAG
yshA-MF	GTCAAATTACAAGAGAAATGTGcgacagcggaattgactc
yshA-MFa	gagtcaattccgctgtcgCACATTTCTCTTGTAATTTGAC
yshA-MR	AAACAACCGTTGACATTTACGG
minC-DF1	CCAGCAGTCCTATAATACGG
minC-DF2.2	ttaaatgagcGGTCTTCACAATATTCACCTC
minC-DR1.2	tgtgaagaccGCTCATTTAAGACCTGATCTA
minC-DR2	CTGATTGGGTAggatccgcGCTATTTGGCTTCAAAAGCG
minC-DR2a	CGCTTTTGAAGCCAAATAGCgcggatccTACCCAATCAG
minC-MF	TCATGTGTTAAATCGCTCCCcgacagcggaattgactc
minC-MFa	gagtcaattccgctgtcgGGGAGCGATTTAACACATGA
minC-MR	TGGACTAACATTGCATCTGG
ugtP-DF1	TGGCGATTGAAATCCAGATG
ugtP-DF2.2	gcactttggcGGTATTCAAGTAAATTCACCTC
ugtP-DR1.2	cttgaataccGCCAAAGTGCTATCGTAATG
ugtP-DR2	CTGATTGGGTAggatccgcgTGCAAAAGGCAGCAACGAGC
ugtP-DR2a	GCTCGTTGCTGCCTTTTGCAcgcggatccTACCCAATCAG
ugtP-MF	GTGCTTCTGATCATGGCAGGcgacagcggaattgactc
ugtP-MFa	gagtcaattccgctgtcgCCTGCCATGATCAGAAGCAC
ugtP-MR	CCAAGAGTCAAATCCGATTG
minJ-DF1	CGATCTTCATGTCAGCCAGC
minJ-DF2.2	cgtcttcacgAACAGACACTATCTCTCACC
minJ-DR1.2	agtgtctgttCGTGAAGACGAAGCAGTCGC
minJ-DR2	CTGATTGGGTAggatccgcGGTCAATTACACCAATGTCG
minJ-DR2a	CGACATTGGTGTAATTGACCgcggatccTACCCAATCAG
minJ-MF	GAAGCGCTTCAGCATAACCGcgacagcggaattgactc
minJ-MFa	gagtcaattccgctgtcgCGGTTATGCTGAAGCGCTTC
minJ-MR	CGTTCTGCGCATGTGAGAAC
ezrA-DF1	GCAGCGATTACAGTCGCATG
ezrA-DF2.2	atatgtcagcAATGACAAACTCCATAATGAGC
ezrA-DR1.2	gtttgtcattGCTGACATATCCGCTTAGATA
ezrA-DR2	CTGATTGGGTAggatccgcgAAGGCTTCTTCTGCATATTGTC
ezrA-DR2a	GACAATATGCAGAAGAAGCCTTcgcggatccTACCCAATCAG
ezrA-MF	GACTAGCAACATTCCAGGCAcgacagcggaattgactc
ezrA-MFa	gagtcaattccgctgtcgTGCCTGGAATGTTGCTAGTC
ezrA-MR	AATCCTGCAGCTCATTGACG
clpX-DF1	GCTTCTTCCAACACAAGCCG
clpX-DF2.2	cctcagtgccGAACGAGCATTTTAATTGTCCT
clpX-DR1.2	atgctcgttcGGCACTGAGGTAAGCCAAGA
clpX-DR2	CTGATTGGGTAggatccgcgAAGCCATCGGTTCTACTGAC
clpX-DR2a	GTCAGTAGAACCGATGGCTTcgcggatccTACCCAATCAG
clpX-MF	CTTCCAGAAGATTTGCTCCGcgacagcggaattgactc
clpX-MFa	gagtcaattccgctgtcgCGGAGCAAATCTTCTGGAAG
clpX-MR	AGCGCATTAACTCCAACAGC

noc-DF1	AACGAAGAGAGATGTACCCG
noc-DF2.2	tgcgaatcgtGAATGAATGCTTCATGTACCTA
noc-DR1.2	gcattcattcACGATTCGCATACCAAAATAG
noc-DR2	CTGATTGGGTAggatccgcgAAAAGCCGCATCAGCTGAAG
noc-DR2a	CTTCAGCTGATGCGGCTTTTcgcggatccTACCCAATCAG
noc-MF	GAACACGATTCGCCAGTCACcgacagcggaattgactc
noc-MFa	gagtcaattccgctgtcgGTGACTGGCGAATCGTGTTC
noc-MR	GAAGAAGGCCAATACGAACTC
spx-DF1	CATAAAGATGAAGGCAAACATC
spx-DF2.2	gctaattgagGGCAAACTAATAGATCGTATC
spx-DR1.2	ttagtttgccCTCAATTAGCTTAACTGATCGAA
spx-DR2	CTGATTGGGTAggatccgcGGACAACGTCATCGCAGTTG
spx-DR2a	CAACTGCGATGACGTTGTCCgcggatccTACCCAATCAG
spx-MF	GAAAAAAATGTGTCAATCCAGCgacagcggaattgactc
spx-MFa	gagtcaattccgctgtcGCTGGATTGACACATTTTTTTC
spx-MR	CTTCTCTTAATTGGAAAGAGCG

* matching sequences in capital letters.

Fig A

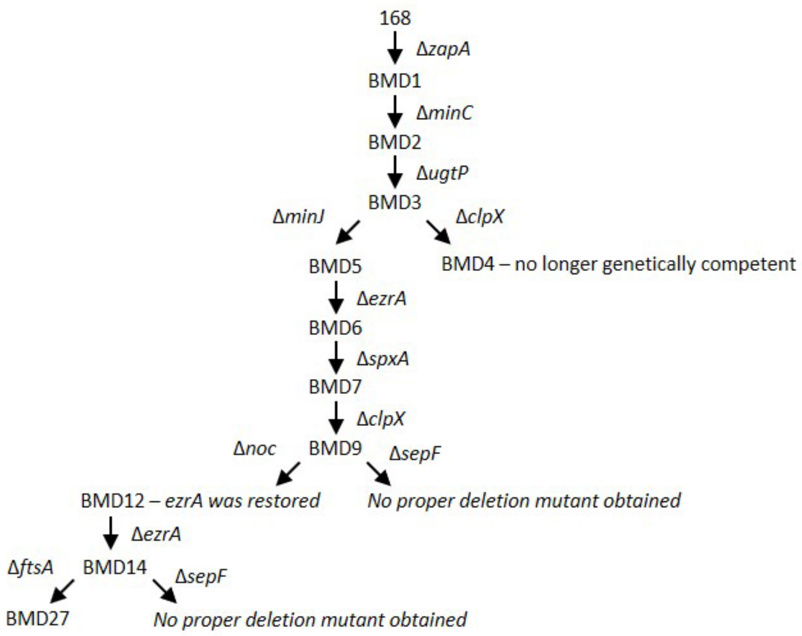

Fig A: Gene deletion pathways.

Overview of the main deletion pathways. After BMD7, further successful deletions were only acquired after repeated transformations, presumably due to the requirement of suppressor mutations. The occurrence of incorrect deletion transformants accounts for the irregular and increased numbering after BMD7.

Fig B

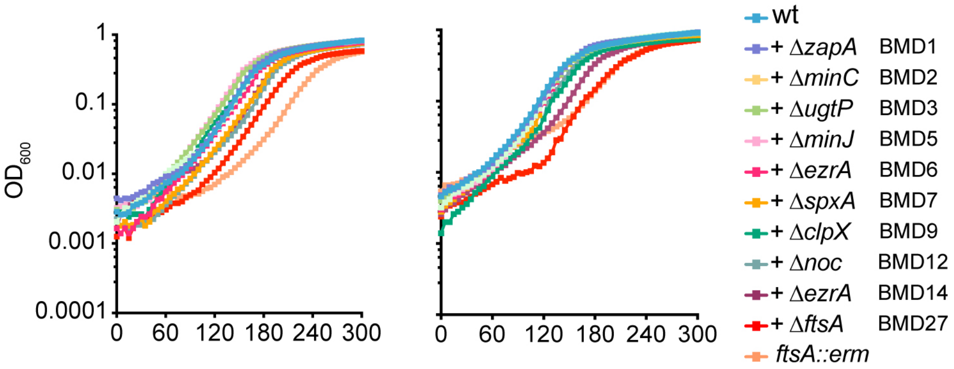

Fig B: Growth of Bacillus minimal divisome strain.

Growth curves of BMD strains grown in microtiter plates at 37 ^{o}C in LB medium supplemented with 1 % glucose and 10 mM $MgSO_4$. Biological replicates related to Fig 2B in main text.

Fig C

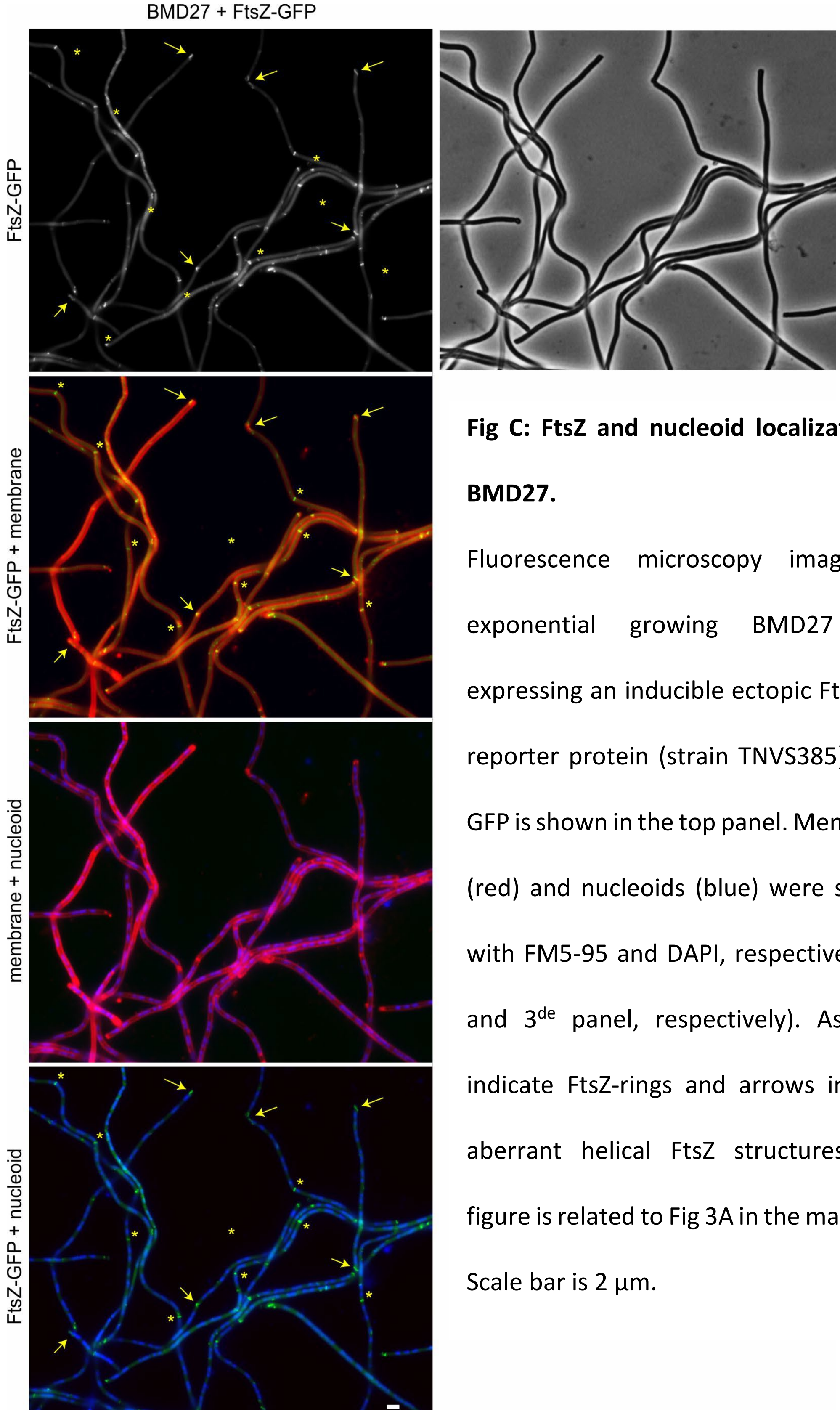

Fig C: FtsZ and nucleoid localization in BMD27.

Fluorescence microscopy images of exponential growing BMD27 cells expressing an inducible ectopic FtsZ-GFP reporter protein (strain TNVS385). FtsZ-GFP is shown in the top panel. Membrane (red) and nucleoids (blue) were stained with FM5-95 and DAPI, respectively (2de and 3de panel, respectively). Asterisks indicate FtsZ-rings and arrows indicate aberrant helical FtsZ structures. This figure is related to Fig 3A in the main text. Scale bar is 2 µm.

Fig D

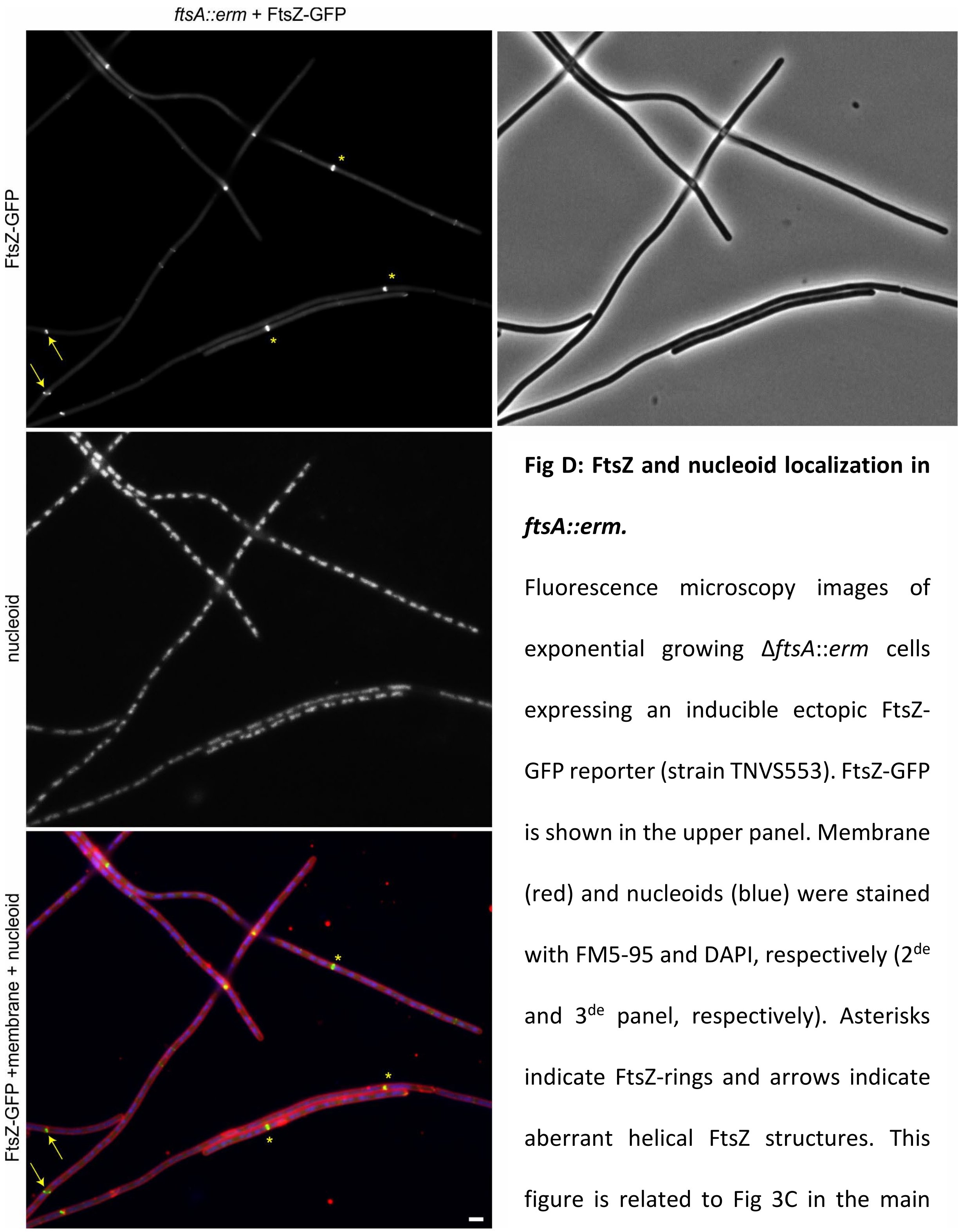

Fig D: FtsZ and nucleoid localization in *ftsA::erm.*

Fluorescence microscopy images of exponential growing Δ*ftsA*::*erm* cells expressing an inducible ectopic FtsZ-GFP reporter (strain TNVS553). FtsZ-GFP is shown in the upper panel. Membrane (red) and nucleoids (blue) were stained with FM5-95 and DAPI, respectively (2de and 3de panel, respectively). Asterisks indicate FtsZ-rings and arrows indicate aberrant helical FtsZ structures. This figure is related to Fig 3C in the main text. Scale bar is 2 µm.

Fig E

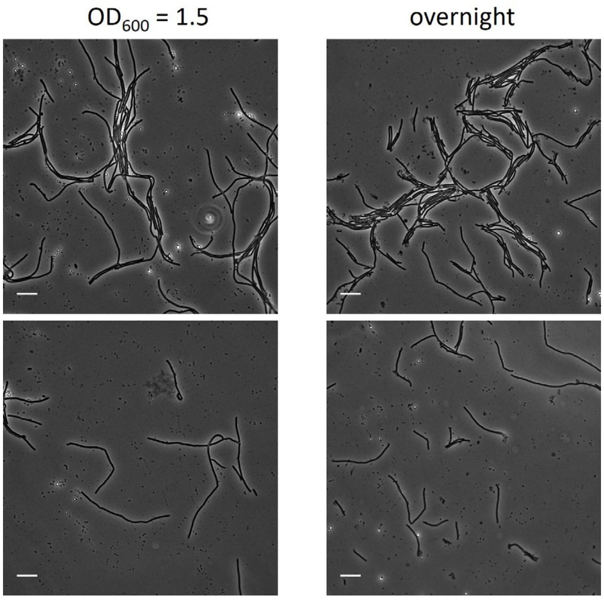

Fig E: BMD27 in stationary phase.

Phase contrast images of BMD27 grown to stationary phase (OD600 = 1.5) and late into the stationary phase (overnight). Bottom frames show more diluted samples. Scale bars 10 µm.

Fig F

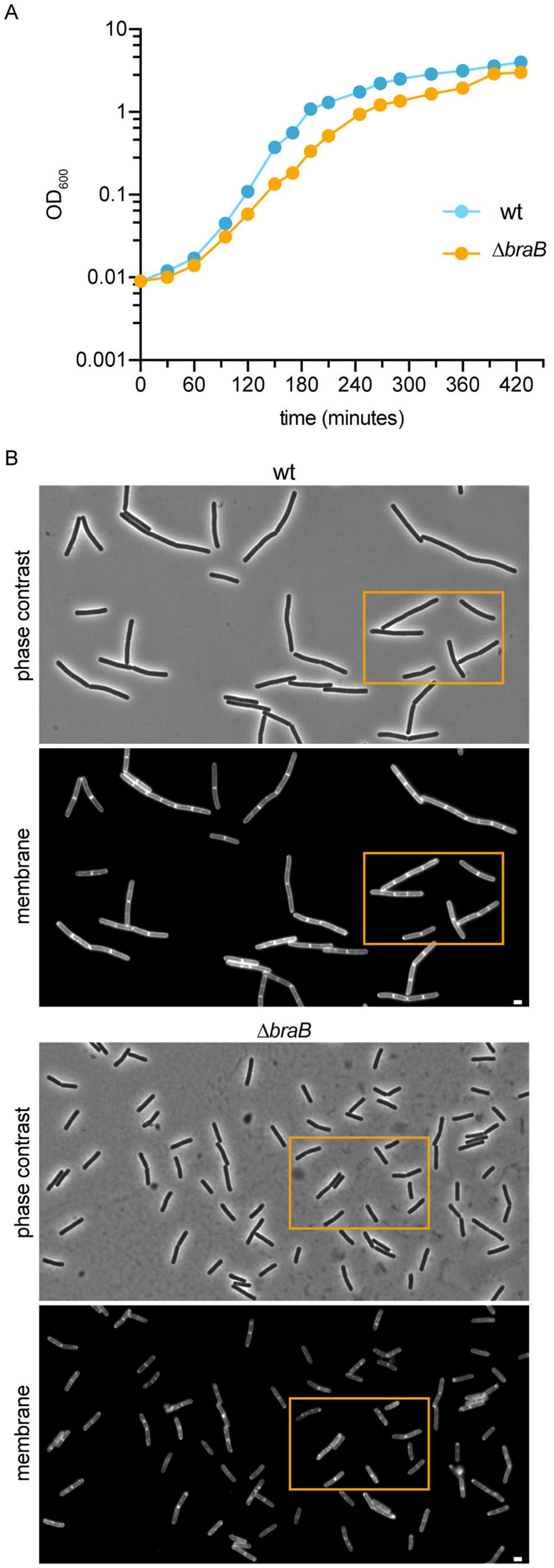

Fig F: Phenotype of the *braB* deletion mutant.

(A) Growth curves of Δ*braB* and wild type cells (wt) grown in LB medium at 37 °C. (B) Aberrant fluorescent membrane pattern in exponential growing Δ*braB* cells (strain TNVS292) compared to wild type cells. Cells were stained with the membrane dye FM5-95. Orange boxed regions are shown in main text Fig 8A. Scale bars are 2 µm.

Fig G

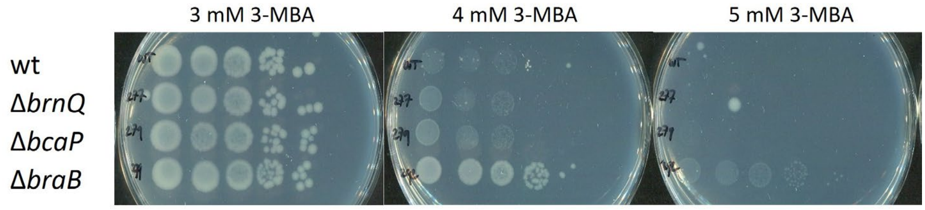

Fig G: Sensitivity of Δ*brnQ,* Δ*bcaP* and Δ*braB* for 3-MBA.

The different strains were grown to OD_{600} 0.5, serial diluted and spotted onto LB agar plates containing either 3, 4 or 5 mM 3-MBA, and grown for 24 h at 37 °C. Strains used: 168 (wild type (wt)), Δ*brnQ* (TNVS277), Δ*bcaP* (TNVS279) and Δ*braB* (TNVS292). Conclusion, inactivation of the other branched chain amino acid transporters BrnQ and BcaP does not increase the resistance to the FtsZ drug 3-MBA.

Fig H

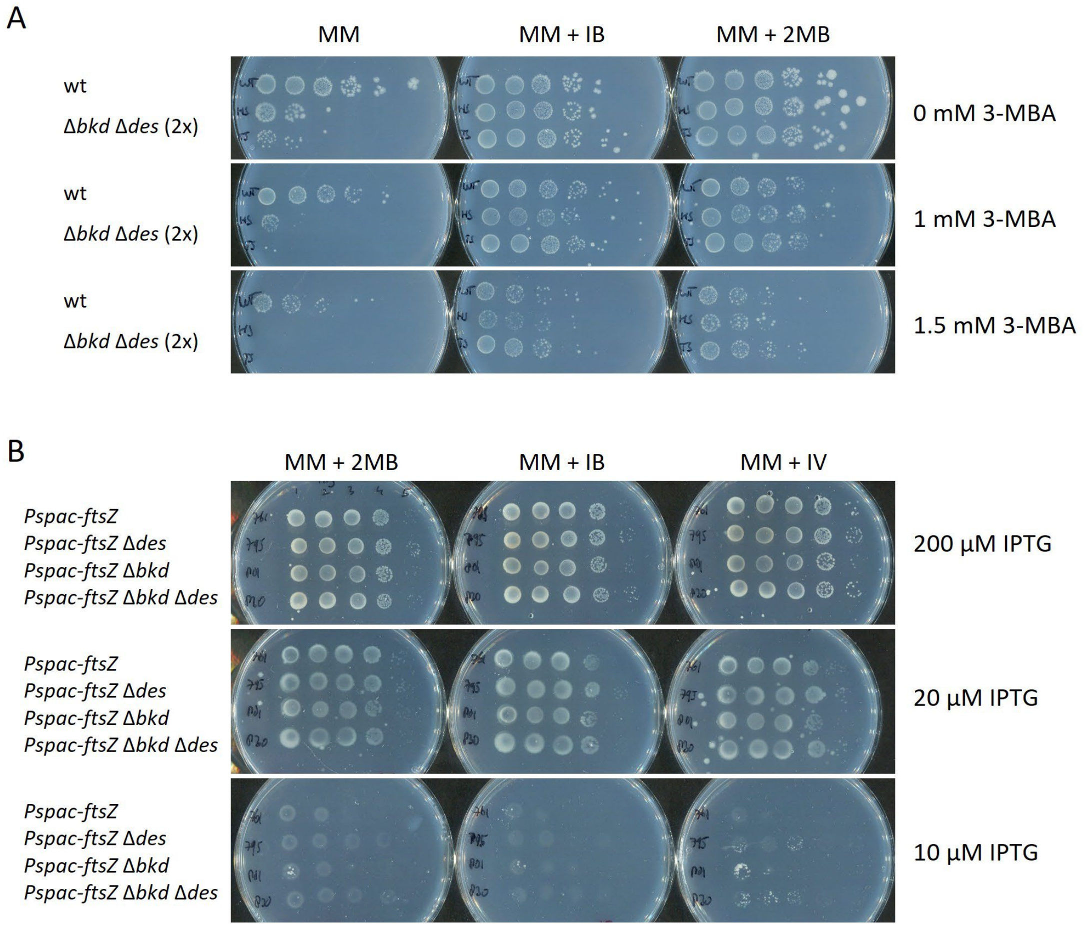

Fig H: Effect of membrane fluidity on sensitivity for FtsZ perturbations.
(A) Wild type (wt) and strain TNVS547 impaired in branched chain fatty acid precursor synthesis (Δ*bkd*) and the phospholipid desaturase Des (Δ*des*) were serial diluted and spotted onto minimal medium agar (MM), containing either 0, 1 or 1.5 mM of the FtsZ inhibitor 3-MBA, without any substitution or with either 0.1 mM isobutyric acid (IB) or 0.1 mM 2-methyl butyric acid (2MB), and grown at 28 °C. The Δ*bkd* Δ*des* strain was spotted twice. Isobutyrate and 2-methyl butyrate are iso- and anteiso-branched chain fatty acid precursors, respectively. The latter precursor will increase the membrane fluidity. Fortified Spizizen minimal medium was used as minimal medium [7]. (B) Effect of reduced FtsZ levels in Δ*bkd* Δdes strains grown with different branched chain fatty acid precursors. In these strain FtsZ is under control of the IPTG inducible promoter *Pspac*. The different strains were serial diluted and spotted onto minimal medium agar (MM), containing either 10, 20 or 200 µM IPTG, and with either 0.1 mM 2-methyl butyric acid (2MB), 0.1 mM isobutyric acid (IB) or 0.1 mM isovaleric acid (IV) another iso-branched chain fatty acid precursor, and grown at 37 °C. Bleomycin was added to the plate since the Pspac promoter has been integrated as single crossover. “Amber medium” was used as minimal medium [8]. Strains used: TNVS 761 *Pspac-ftsZ*, TNVS 795 *Pspac-ftsZ* Δ*des*, TNVS 801 *Pspac-ftsZ* Δ*bkd*, TNVS 830 *Pspac-ftsZ* Δ*bkd* Δ*des*.

REFERENCES

1. Morimoto T, Ara K, Ozaki K, Ogasawara N. A new simple method to introduce marker-free deletions in the *Bacillus subtilis* genome. Genes Genet Syst. 2009;84(4):315-8. Epub 2010/01/09. doi: JST.JSTAGE/ggs/84.315 [pii]. PubMed PMID: 20057169.
2. Ishikawa S, Kawai Y, Hiramatsu K, Kuwano M, Ogasawara N. A new FtsZ-interacting protein, YlmF, complements the activity of FtsA during progression of cell division in *Bacillus subtilis*. Mol Microbiol. 2006;60(6):1364-80. PubMed PMID: 16796675.
3. Zeigler DR, Pragai Z, Rodriguez S, Chevreux B, Muffler A, Albert T, et al. The origins of 168, W23, and other *Bacillus subtilis* legacy strains. J Bacteriol. 2008;190(21):6983-95. Epub 2008/08/30. doi: 10.1128/JB.00722-08. PubMed PMID: 18723616; PubMed Central PMCID: PMCPMC2580678.
4. Gamba P, Veening JW, Saunders NJ, Hamoen LW, Daniel RA. Two-step assembly dynamics of the *Bacillus subtilis* divisome. J Bacteriol. 2009;191(13):4186-94. Epub 2009/05/12. doi: JB.01758-08 [pii]10.1128/JB.01758-08. PubMed PMID: 19429628; PubMed Central PMCID: PMC2698510.
5. Feucht A, Errington J. *ftsZ* mutations affecting cell division frequency, placement and morphology in *Bacillus subtilis*. Microbiology. 2005;151(Pt 6):2053-64. doi: 10.1099/mic.0.27899-0. PubMed PMID: 15942012.
6. Koo BM, Kritikos G, Farelli JD, Todor H, Tong K, Kimsey H, et al. Construction and Analysis of Two Genome-Scale Deletion Libraries for *Bacillus subtilis*. Cell Syst. 2017;4(3):291-305 e7. Epub 2017/02/13. doi: 10.1016/j.cels.2016.12.013. PubMed PMID: 28189581; PubMed Central PMCID: PMCPMC5400513.
7. Gohrbandt M, Lipski A, Grimshaw JW, Buttress JA, Baig Z, Herkenhoff B, et al. Low membrane fluidity triggers lipid phase separation and protein segregation in living bacteria. EMBO J. 2022;41(5):e109800. Epub 2022/01/18. doi: 10.15252/embj.2021109800. PubMed PMID: 35037270; PubMed Central PMCID: PMCPMC8886542.
8. Bohorquez LC, de Sousa J, Garcia-Garcia T, Dugar G, Wang B, Jonker MJ, et al. Metabolic and chromosomal changes in a Bacillus subtilis whiA mutant. Microbiol Spectr. 2023;11(6):e0179523. Epub 2023/11/02. doi: 10.1128/spectrum.01795-23. PubMed PMID: 37916812; PubMed Central PMCID: PMCPMC10714963.
