# Supplementary material for: Minimization of the Bacillus subtilis divisome suggests FtsZ and SepF can form an active Z-ring, and reveals the amino acid transporter BraB as a new cell division influencing factor
Source: PLoS Genet. 2025 Jan 27;21(1):e1011567. doi: 10.1371/journal.pgen.1011567 (PMC11790237; doi:10.1371/journal.pgen.1011567)
Supplement: Attachment — Submitted filename: response to reviewers.pdf [file pgen.1011567.s005.pdf]

Response to reviewers' comments

We would like to thank the reviewers for their efforts and positive comments. For clarity, we have provided our answers in *blue*.

Reviewer #1:

This interesting paper by Gulsoy et al. seeks to identify the minimal set of genes required for cell division of a Gram-positive walled bacterium (the model Bacillus subtilis). In E. coli and B. subtilis model systems, cell division consists of a setup stage in which FtsZ polymers form a midcell Z ring by attaching to the cytoplasmic membrane via adaptor proteins, a recruitment stage wherein the septum-synthesis enzymes are brought to the Z ring, followed by a septum synthesis stage. In this study, the authors tested the minimal gene requirements for formation of the first stage--an active cytokinetic Z ring—by sequential deletion of conserved genes involved in cell division, starting with the least crucial and ending with the more crucial genes. After trying out several schemes for sequentially deleting the genes, they found that most of the conserved early stage division genes could be deleted, including ftsA and ezrA, and cells left with only sepF and ftsZ in this first stage were able to divide, albeit poorly. This reliance mainly on SepF and FtsZ is perhaps not too surprising, as Gram-positive Actinobacteria carry only SepF and FtsZ as their essential cell division genes (mentioned on lines 453-456) and not the others. So, the authors basically forced B. subtilis to become Actinobacteria in terms of cell division, with Actinobacteria being closer to a minimal cell than B. subtilis.

Many of the gene deletions nevertheless gave rise to suppressors, some of which were known to regulate cell division. The authors did a nice job in carefully identifying the suppressors and testing their effects, although most of them did not lead to any obvious reason for why they arose in response to the gene deletions. There was one suppressor with strong effects on cell division, however: a loss of function allele in braB, which encodes a branched chain amino acid transporter. A deletion of braB (which does not affect the uptake of branched-chain amino acids) was notable in that it resisted the effects of an anti-FtsZ compound, 3-MBA, and allowed normal cell division to occur at artificially low levels of FtsZ. These traits, along with the ability of the braB knockout mutant to divide at shorter cell lengths than WT cells, and the lack of BraB-GFP localization at division septa, suggested that the activity of BraB somehow acts as an indirect inhibitor of cell division. Notably, cell membranes in a braB mutant had different lipid composition and higher overall fluidity, including visible membrane domains of higher fluidity. It is suggested that this higher membrane fluidity in the absence of BraB somehow promotes cell division. Although it is exciting to have found a new factor in promoting cell division in B. subtilis, it was also frustrating that so little was gleaned from the suppressor findings, especially as the links between branched chain amino acid transport and membrane fluidity are tenuous. Otherwise, the paper is well written and clear, and provides some new insight into requirements for cell division proteins in B. subtilis. The Discussion is thorough and thoughtful.

We would like to begin with thanking the reviewer for the very extensive and useful feedback.

Specific comments:

Lines 79-80: There are a few exceptions to this. Chlamydia, which has a cell wall, uses MreB to divide instead of FtsZ. And Planctomycetes lack FtsZ.

This is indeed true, but we feel that it hampers the reading when we have to discuss these exceptions in detail. To accommodate the reviewer we now begin this paragraph with "Generally," (line 75).

Line 92: replace "also displays some inhibitors activity when it comes to" with "can inhibit"

Changed as suggested.

Line 102: should be "proteins" in both instances.

Corrected.

Line 105: replace “can amount to” with “is approximately”
Changed as suggested.

Line 114: replace “couple” with “pair”
Changed as suggested.

Line 148: replace “in” with “map to”
Changed as suggested.

Line 149: should be “led”
Corrected.

Line 207: Do the authors have an explanation for why multiple minicells form at the expense of division septa elsewhere? Is there some kind of positive feedback occurring?
No, we do not have a good explanation for this phenomenon. Maybe there is indeed a kind of positive feedback occurring based on cooperative assembly of division machinery components, but this is speculation.

Line 221: E. coli lacking Min and SlmA also still show a preference for Z rings between nucleoids, so this may be a conserved trait (PMID: 25101671).
This is a good point and we have now included this reference and the E. coli situation (line 220-222).

Line 301 (and 1085) should be “IPTG”
Corrected.

Lines 306-307: lower FtsZ concentrations should have a different effect from high 3-MBA concentrations, as the latter should stop FtsZ polymer treadmilling, unlike the former. Perhaps FtsZ-mediated septum formation in B. subtilis (which PonA contributes to) is somewhat treadmilling-independent, while it absolutely requires a minimum number of FtsZ molecules?
This is an interesting suggestion that we will remember for future studies, however, at the moment it is too speculative to mention here in the manuscript.

Lines 319-320 and Fig. 7C: How do the authors know for sure that there was no accumulation of BraB-GFP at division sites? Were the levels of fluorescence at septal membranes compared with those of a bona fide division protein or a known non-division membrane protein?
The fluorescent signal is comparable to what is found when using a fluorescent membrane dye, which gives always a slightly higher signal at septa because of the presence of more membrane material, i.e. a double membrane together with the fact that these double membranes are in the line of sight. We mention this now in lines 328-330.

Line 373-375: although FtsA-FtsN interaction is important for FtsN’s recruitment and subsequent activation of the septum synthesis enzymes, other key division proteins such as FtsQLB can be recruited independently of FtsN (and FtsA-FtsN interactions). Indeed, FtsN can be partially or completely bypassed with hyperfission alleles.
True, but in the minimal divisome mutant only SepF is left, therefore we speculate here that SepF directly interacts with the late cell division proteins.

Line 394: replace “one in” with “one maps to”
Changed as suggested.

Line 437-438: this is not a full sentence

We have reformulated the sentence.

Line 453: should be "positioning"
Corrected.

Line 452: are FtsZ and SepF really "sufficient"? Other unknown proteins may play a nonessential role that may become essential in the BMD27 mutant.
This can indeed be the case, therefore we have added now a disclaimer ",although we cannot exclude that some unknown protein might have become essential for cell division in the minimal divisome mutant" (line 463-464).

Line 460: Z rings also still are biased to form between nucleoids of E. coli cells that lack the nucleoid occlusion and Min systems (Bailey?).
We have now added the references to the E. coli system, where the same mechanism is postulated.

465: synthesize
We do not understand this remark (it is synthetic cell and not synthesize cell)

468: Some Gram negative cells such as the rhizobiales grow via polar growth as well.
Here we refer specifically to Actinomycetes because there cell division mode have been thoroughly investigated unlike other bacteria with polar growth, and they are related Gram-positives.

Reviewer #2:
The manuscript by Gulsoy et al describes the construction of a Bacillus subtilis strain lacking eight conserved cell division proteins, with the aim of identifying the minimal divisome machinery. The work indicates that FtsZ and SepF are key to form an active Z-ring and proposed a new role for BraB in cell division. This is a considerable effort, of interest to the community studying bacterial cell division.

General comments
Paragraph starting in line 177 – Authors mention that they tried different sequences of successive deletion of division genes but most resulted in a lethal phenotype. A supplementary figure with the failed paths should be included in the paper, as these can be informative regarding negative genetic interactions and also inform other researchers on paths that are not worth to follow.
Firstly, we would like to thank the reviewer for the effort and useful feedback.

With regard to the specific comment, we should clarify that we have not systematically tried all combinations, and that our formulation in line 178 might have given this impression. We have adjusted the sentence to make this more clear. We have now included an overview of the different deletion pathways in a new figure, Fig. S1, in the SI. The difficulties with obtaining the required deletions emerged after MBD7 and required multiple transformation attempts, presumably because further deletions requires the presence of suppressor mutants, as we describe later in the manuscript.

Line 205 (and various places in the manuscript) – mention strain name. This helps the reader in case they need to check the genotype
We have now added strain names in the text, except for the part that describes Fig. 6, in which a large number of mutants have been tested. Mentioning all these mutants will reduce the readability of the text.

Line 211 – The described results were not obtained with strain BMD27, as mentioned in the text, but with a derivative of this strain expressing FtsZ-GFP.

This has now been adjusted.

Line 215 – stating "approximately half of the Z-rings…" instead of "the majority of Z-rings" would be more in line with the data.
This text has been adjusted as suggested.

Section starting on line 226 and Fig 5 – There are various suppressor mutations in BMD strains that appear and disappear on the genome of sequentially made strains. For example, the gltP mutation is present in BMD2, absent in BMD 2,3 and 5, and present again in BMD6. Then absent in BMD 7 and 9, but present again in BMD 12, 14 and 27. How do author explain this if the strains were made sequentially? This is a key point that has to be explained.
This is a good point. The appearance and disappearance of mutations is caused by the fact that we use chromosomal DNA from single knockout strains for transformation. Mutations in the DNA-receiving strain can be restored by means of an extra double crossover event, as is the case for the restoration of the ezrA deletion in the BMW9 strain after transformation with DNA from a single Δnoc mutant strain. We have explained this now better in line 193 and lines 275-275. However, in case of gltP something else is going on since this is a spontaneous 2 bp deletion (GCCCA to GCC) reappeared twice, in BMD6 and BMD14. We do not have an explanation for this and mention this now in line 277-279.

Line 311-312 – referring to deletion of braB, authors state: "there is no clear reason why this would cause an increased resistance to 3-MBA and lower FtsZ levels". This sentence implies "there is no clear reason why this (deletion of braB) would cause (…) lower FtsZ levels", which was not shown. Do authors mean "increased resistance to 3-MBA and to lower FtsZ levels"?
The latter is indeed the case. We have now corrected this sentence.

Line 418 - briefly explain why DivIB was not considered here.
Unfortunately, we do not understand what the reviewer means here, since this part is about MinCD and also lipids, and we do not know of any specific paper related these to DivIB.

Figure 2 – it is difficult to distinguish some of the colours. Maybe ordering colours in a gradient would help? Also, in the labels of the graphs in this figure "Dnoc" should be indicated as "Dnoc + EzrA". Otherwise the reader maybe misled. In the legend of this figure, spx should be mentioned, so that it is clear that it is not a division protein
We have tried to use colour gradients but this did not make it more clear, unfortunately. As suggested, we have changed "Δnoc" to "Δnoc ezrA" in the figure (Fig. 2B and C), and we have indicated that spxA is not a cell division gene in the figure legend.

Figure 7 B – add strain names to the right of the images. Also in both panel B and C there is a strain labelled DBraB+braB-gfp, but in the legend two different strains are mentioned. This may be confusing to the reader. The title of this figure can be improved.
We agree that the legend is, indeed, insufficiently clear. Therefore, we have rephrasing the legend and clearly indicate the strains. We have also changed the title to "BraB-GFP localization", which reflect more precisely the experiment.

Minor comments/typos
Lines 101, 102 and 108 – replace "the late cell division protein" by "late cell division proteins"(plural) or "the late cell division protein complex"
Corrected.

Line 134 – Minc – "C" should be capital letter.
Corrected.

Line 149 – "we also found an unknown suppressor mutation" – all suppressor mutations are unknown before genome sequencing. Maybe "unexpected suppressor mutation"?
Changed as suggested.

Line 200 – remove comma after BMD27
Removed.

Line 213 – do authors mean dissect or bisect?
Bisect. Corrected.

Line 974 – remove "a" before "more details"
Corrected.

Line 1045 – strains should not be plural
We feel that in this case plural is more accurate.

Line 1047 – "seems to bisect" instead of "seem to dissect"?
Yes, corrected as suggested.

Figs S2 and S3 – Phase contrast images are too small. They could be shown to the right of the bottom fluorescence image.
Changed as suggested.

Reviewer #3:
This paper sets out to determine the minimal set of cell division genes that are essential. They cleverly sequentially delete as many cell division genes as possible, finding that only FtsZ and SepF are essential. This is the first study in a cell wall containing bacterium to identify this minimal set. This is the strongest part of the manuscript. The finding that loss-of-function mutations of BraB suppress the cell growth defect of the minimal FtsZ, SepF mutant are interesting, but the conclusions about how this protein may influence cell division are unclear. I detail a number of comments would strengthen the manuscript below:

1. Line 207, page 10, indicate that multiple polar minicells are observed in Figure 3B. This statement needs to be removed on better data presented. It was not possible to see these minicells in figure 3B, possibly because the membrane stain appears so weak.

We would like to begin with thanking the reviewer for carefully reading our manuscript and the useful feedback. Concerning the membrane stain in Fig. 3B, on our screens the membrane stain shown in the top panels (labelled "nile red") are clearly visible. However, we agree that these cell pole deformations do not look like classical minicells and we have rephrased the sentence to: "The absence of 8 cell division proteins resulted in long filamentous cells with less Z-rings and polar deformation that seem to be caused by aberrant polar divisions" (line 209).

2. Line 209, page 10, conclude that the BMD27 cells are only delay in cell division because the filamentous cells divided and became smaller in stationary phase. This is an important point, but the data is not shown. Please show this data in the supplemental material.

We have now added phase contrast images of BMD27 in the stationary phase in the SI (Fig. S5).

3. Lines 221-224, page 10, indicate that FtsA is required for midcell Z-ring formation, and that this is likely due to the formation of very long filamentous cells rather than a regulatory role. However, as shown in Figure 2C, the BMD14 mutant, which does form midcell Z-ring, is similarly long and filamentous as the FtsA mutant. This would argue for a more regulatory role for FtsA. Please address this confusing data in the text of the manuscript.

This is a good point, and we have rephrased this now to: "However, removing FtsA seems to abolish midcell selection, which was also the case for a ftsA single deletion mutant (Fig. 4). Since these mutants forms cells with a comparable length as the BMD14 mutant containing FtsA (Fig. 2C), it seems like FtsA has some regulatory role in FtsZ ring positioning." (line 226-228).

4. Line 319, page 14, indicated that the BraB-GFP signal does not accumulate at division sites. However, as shown in Figure 7C, there does appear to be a central focus of BraB-GFP. The presence of this focus should be address in the text of the manuscript.

We have looked at the localization of BraB-GFP extensively, with the hope to see some cell division site recruitment, but we must conclude that we cannot find a positive association with cell division sites. During cytokinesis a double membrane is formed at the middle of the cell and this can show up as a slightly more fluorescent band, also because of the perpendicular viewing angle, but this is not the same as an active recruitment to cell division sites. We clarify this now in lines 328-330.

5. Line 397, page 19, indicated that inactivation of other branch chain amino acid transported did not affect resistance to 3-MBA, as inactivation of BraB did. This data is not shown, but it is important enough to show in the supplemental results.

We have added this data now to the SI (new Fig. S7).

6. Line 399, page 19, states that the BraB-GFP fusion showed no preference for the cell division sites. This line should be changed as the data does not seem to support this conclusion.

We do not think that this is the case as explained in lines 328-330. See also our reply to previous remark nr 4 of the reviewer.

7. One interesting question about BraB is whether it is the loss of its enzymatic activity that is required to suppress the loss of cell division genes. As of now, there is a correlation between changes in membrane composition and suppression of cell division defects, but this could be due to a second function of BraB that is independent of its role in forming branched-chain fatty acids. It is not clear whether there is a particular amino acid change that can be made that would disrupt it activity as a symporter, but testing such a mutant could add significantly to your model of how BraB affect cell division.

We agree with this, and it is a good suggestions for future studies aimed at elucidating the function of BraB in cell division.

8. Line 431, page 20, indicates that you have data showing that changing the membrane fluidity, independently of BraB inactivation, did not result in cell division suppression. This data is not shown, and again this is an important point that should be shown in the figure. This evidence, which is only brought up in the discussion, further argues the need to understand whether the enzymatic activity of BraB is important.

We have now included this data in Fig. S8 of the Supplementary Information

9. On line 102, on page 5, please indicate to which late cell division proteins you are referring.

We describe these proteins in the previous sentences, but we have slightly reformulated this part to make it more clear (lines 95-97).

10. Line 287, page 13, indicated that strains with deletion mutations were constructed. This should

indicate whether this was done in the background of a BMD mutant or the wild-type background.
This should indeed be clarified. We now mention that these mutations were introduced into the B. subtilis wild type background (line 295).

11. On Figure 2B, the results are hard to see because the lines are so close to each other and the colors so similar to each other. Adding numbers for the growth rate would help make the data in this figure more interpretable.
The purpose of this figure is to show that the growth rate is not greatly affected and even in the most filamentous mutants still reach acceptable optical densities in time. We feel that adding growth rates does not facilitate interpretation of this figure.

12. Figure 3A and 3C should address why the membrane stain shows as a couple of spots and not the length of the cell.
We have the feeling that this might be a screen or pdf print issue, since we can clearly see the complete membrane in Fig. 3, and this problem is not raised by the other reviewers. We assume that with the higher resolution images provided with the revision, this problem will be resolved.

13. Figure 8C, it is not clear from the legend how the data in figure 3C was calculated. A formula should be added for clarity
The ratio was calculated from the data shown in the table in Fig. 8B. We mention this now in the legend of the figure.
